# Measured and Perceived Body Weight Status of Women in the Peruvian Amazon

**DOI:** 10.3390/medicina56080375

**Published:** 2020-07-26

**Authors:** Sophie Budge, Agnieszka Jaworowska

**Affiliations:** 1 Cranfield Water S1ience Institute, Cranfield University, College Road, Cranfield MK43 0AL, UK; s.g.budge@cranfield.ac.uk; 2 School of Science, Faculty of Engineering and Science, University of Greenwich, Central Avenue, Chatham ME4 4TB, UK

**Keywords:** obesity, overweight, weight perception, Peru, Latin America

## Abstract

*Background and objectives:* The prevalence of obesity among adults has reached epidemic proportions in Latin America, placing large demands on health care systems. Research suggests cultural differences in body weight perceptions may be a barrier during the implementation of weight-loss strategies. The aim of this study was to examine the prevalence of weight misperception in Peruvian women and evaluate contributing factors. *Materials and Methods:* A total of 236 women were recruited in San Martín, northern Peru. Participants’ socio-demographic characteristics and attitudes towards their weight and health were collected. Self-perception of weight status was assessed with a 10-point scale and compared with measured body mass index (BMI). Multiple logistic regression analysis was conducted to identify factors associated with underestimation of weight status. *Results:* A total of 65.2% of women were classified as overweight/obese by BMI, but only 15.2% perceived themselves so. A total of 70.4% of women underestimated their weight status and no incidence of overestimation was reported. Overweight and obese women were more likely to underestimate their weight status than normal weight women (OR (Odds Ratio): 34.24, 95% CI (Confidence Interval): 11.55–101.45; OR: 42.06, 95% CI: 11.17–158.32, respectively). Women who underestimated weight status felt more comfortable with their weight (59.3% vs. 20.6, *p* < 0.001) and agreed a large stomach is a sign of good health (40.7% vs. 5.9%, *p* < 0.001) versus those who correctly estimated. *Conclusions:* Underestimation of weigh status was highly prevalent and associated with unhealthy beliefs. Future public health programs must be culturally sensitive and tailored to specific groups within the population.

## 1. Introduction

The prevalence of overweight and obesity among adults has reached epidemic proportions worldwide, placing large demands on health care systems which must manage the burden of related non-communicable disease, such as diabetes, cardiovascular disease and some cancers [1]. The global surge in excess weight has occurred rapidly: the prevalence of obesity increased from 3.2 to 10.8% in men and from 6.4 to 14.9% in women between 1975 and 2014 [2]. However this pattern of increase is heterogeneous across the world, with rates of obesity currently rising much more rapidly in low- to upper middle-income countries (LMICs) than in developed countries [2]. In Latin America, almost one quarter of the population is obese [3], with a predicted prevalence of 43.6% by 2030 [4]. Similar trends are observed in Peru, with overweight and obesity prevalence at 43.6% and 26.9%, respectively [5]. Significantly, a greater proportion of excess weight appears among the adult female population, with an estimated 24.2% obese vs. 15.2% of men [6].

Across Peru, a rapidly changing economic climate and urbanisation have led to profound changes in diet and lifestyle [7], where the traditional rural diet—low in fat and processed sugar and high in fibrous pulses and root crops—has been replaced by foods high in simple, refined carbohydrates and fats. Given the pivotal traditional roles that women hold in the selection, buying and preparation of food for their partners, children and often entire families [8], the increased prevalence of excess weight among women is of particular concern. Data from 2012 suggested that obesity was higher among women in urban areas (16.2% [95% CI 15.2–17.2] versus 8.4% [95% CI 7.5–9.3]), increasing at higher levels of wealth. However, it is noted that this varies by level of urbanisation [9].

Though dietary and lifestyle factors are significant in shaping population weight status, differing levels of cultural acceptance and perceptions of body size also play a role [10]. Increases in overweight across regions is associated with a decrease in correct perceptions of body status [11]—documented both throughout Latin America [12,13] and in Peru [14,15]. Perception of weight is influenced strongly by socio-cultural norms, values, traditions, gender, age, ethnicity and socio-economic status [16,17,18]. It has been also reported that in developing countries, which have until recently mainly focused public health strategies on infection and undernutrition, excessive body weight may be seen as a sign of wealth [15].

Importantly, correct perceptions of body size in those of excess weight strongly correlate with attempts to lose weight and behavioural change. Thus these socio-cultural determinants may be a considerable barrier during the implementation of strategies that target weight loss [19]. An improved understanding of factors associated with misperception may provide insights into opportunities for intervention and to develop public health programmes to tackle obesity. Of particular importance is describing this phenomenon among women – the primary caregivers responsible for the family’s diet and health, whom are also experiencing the greatest increases in excess weight [19,20]. Whilst weight misperception is well documented in developed countries, in Peru, studies on self-perception of weight status and its determinants are limited. This study therefore aimed to compare self-perceived weight status and measured BMI (Body Mass Index) in a sample of Peruvian women to understand and describe the discrepancy between subjective and objective weight and to assess factors associated with weight misperception. 

## 2. Materials and Methods

### 2.1. Study Sites and Sampling Methods

This study was conducted in the department of San Martín, northern Peru, in Moyobamba, the capital city of the region (approximately 43,000 residents) and Yantaló, a rural village (approximately 2800 residents) [21] between May and June 2014. Within Moyobamba, maps of the city were used to group neighbourhoods. Neighbourhoods were listed, numbered, and selected at random using a lottery method. Within the selected neighbourhoods, starting at one end of each street, the study team visited every other house. Women who lived in the house over the age of 18 were invited to participate. In Yantaló, due to the rurality and small size of the village, researchers employed a non-probability method, which purposively selected for women who self-reported as aged over 18. Starting at the main plaza, researchers systematically took in turn each main road leading outward and each connecting side road. Again, researchers visited every other house and eligible women were invited to participate. Pregnant women, visitors and tourists to the area were excluded from participation. A total of 236 women agreed to participate in the study. Two participants were underweight (BMI < 18.5 kg/m^2^) and four declined consent for anthropometric measurement. The final sample totalled 230 women (*n* = 116, Yantaló; *n* = 114, Moyobamba).

### 2.2. Questionnaire—Attitudes towards Weight and Self-Perception of Weight Status 

A short questionnaire assessed participant socio-demographic characteristics (age, level of education, employment status, marital status and the number of people in the household). In addition, respondents’ attitudes towards their weight and health status were evaluated with six questions adapted from a previously validated questionnaire [22]: ‘My health and my weight are very important to me’; ‘I believe it’s my responsibility to look after my weight’; ‘I feel comfortable with my weight’; ‘I often have problems with controlling my weight’; ‘A large stomach is a sign of good health and wellbeing’; and ‘In my opinion, overweight makes people live longer than thin people’. Possible answers were ‘Yes’, ‘No’ or ‘Slightly/A bit’. To assess self-perception of body weight a 10-point linear scale was used which ranged from ‘underweight’ (1), through ‘normal weight’ (5), to ‘obese’ (10). Participants were asked to mark which point on the scale that they felt corresponded with their current weight status. 

### 2.3. Anthropometric Measurements 

Participants’ height, weight, and waist circumference were measured and recorded by two trained researchers. Weight and height were measured on subjects wearing light clothes and no shoes with an inflexible measuring tape against a wall measured to the nearest 0.1 cm and with a calibrated electronic scale measured to the nearest 0.1 kg, respectively. All measurements were taken in triplicate and an average value was used for analysis. Body mass index (BMI) was calculated as weight (kg)/height (m^2^), and normal body weight was defined as BMI ≥ 18.5–24.99, overweight ≥ 25–29.99, obesity ≥ 30 [23]. Waist circumference (WC) was measured according to the World Health Organisation protocol [24] at the midpoint between the last palpable rib and the top of the iliac crest. The cut-off point of ≥ 80 cm for women defined central obesity, according to the International Diabetes Federation waist circumference cut-off points for Ethnic Central and South American populations [5,25].

### 2.4. Analysis

#### 2.4.1. Study Variables: Measured and Perceived Weight

For analysis, participants were grouped into three categories of self-perceived body weight status according to their score on the 10-point scale: ‘underweight’ (1–4) ‘normal’ (4.1−6) ‘overweight’ (6.1−8) and ‘obese’ (8.1–10). Based on the comparison of self-perception of body weight and measured BMI, accuracy of weight estimation was calculated by categorising participants into three categories: ‘underestimated’ (participants who perceived themselves as lighter than their measured BMI), ‘correctly estimated’ (self-perception matched BMI) or ‘overestimated’ (participants who perceived themselves as heavier than the corresponding BMI).

#### 2.4.2. Statistical Analysis 

Statistical analysis was performed using SPSS^®^ (version 26.0, IBM, Chicago, IL, USA). A *p*-value ≤ 0.05 was considered significant. Frequency statistics described respondents’ socio-demographic characteristics and anthropometry. Differences among women living in rural and urban locations and between women who underestimated and correctly estimated their weight status were assessed using Pearson’s chi-squared test for independence. Binary logistic regression models evaluated the association between accuracy of weight estimation and socio-demographic factors, BMI and WC. Odds ratio (OR) and the 95% confidence interval (95%Cl) were presented. 

## 3. Results

### 3.1. Participant Characteristics

Sample characteristics are summarised in Table 1. Most women were aged 18−29 years (39.6%), with a slightly higher prevalence of younger respondents in the urban than rural population (50.0% and 29.3%, respectively). Education levels were higher in the urban than rural community where 11.4% and 33.6%, respectively had no education and 33.3% and 4.3% had achieved a technical career or apprenticeship. The highest prevalence of any type of employment was in the urban sample at 67.5% vs. 29.3% in rural, although this highlights a discrepancy in defining the term ‘employment’ in rural areas, where the traditional role of housewife (‘ama de casa’) is the principal role and occupation for most women. Almost two-thirds of women were classified as either overweight or obese by measured BMI, but only 15.2% perceived themselves as overweight/obese. The rural population exhibited higher levels of overweight than the urban (49.1% vs. 37.7%), but obesity prevalence was similar in both groups (22.4% vs. 21.1%). Slightly higher prevalence of abdominal obesity (waist circumference ≥80 cm) was observed in the rural than the urban group (86.2% vs. 76.3%, respectively).

### 3.2. Association between Self-Perceived Weight, Accuracy of Weight Estimation and Measured BMI 

In terms of self-perceived weight, a very high level of misclassification was evident, with a significant trend towards underestimation of weight (*p* < 0.001; Table 2). Two-thirds of women underestimated their weight (70.4%, Table 1), with a higher level of underestimation among overweight (90.0%) and obese (90.0%) than normal weight women (33.8%; *p* < 0.001; Table 2). There was no incidence of overestimation among the studied women. Similarly, accuracy of weight estimation differed across BMI categories (*p* < 0.001; Table 2), with normal weight women estimating most accurately (66.2%). Only 10% of overweight and obese women were able to correctly estimate their weight status. In addition, a higher prevalence of underestimation was seen in the rural (80.2%) than the urban community (60.5%; *p* < 0.005; Table 1). 

### 3.3. Association between Accuracy of Weight Estimation and Attitudes towards Weight and Health Status 

Associations between survey responses and accuracy of weight estimation are presented in Table 3. The majority of women across both sample sites considered their health and weight important to them, regardless of whether they underestimated or correctly estimated their weight status (81.5% vs. 91.2%). However, only 34.6% of those who underestimated their weight believed that it was their responsibility to look after their weight, in contrast to 97.1% of women who correctly estimated. A significantly higher proportion of women who underestimated their weight status felt comfortable with their bodies in comparison to those who correctly estimated (59.3% vs. 20.6%, respectively, *p* < 0.001). Similarly, a significantly larger proportion of underestimators said they did not experience problems with controlling their weight compared with those who correctly estimated weight (51.2% vs. 26.5%; *p* < 0.001). A total of 54.4% of women who correctly estimated weight agreed that they had problems with controlling their weight (vs. 26.6% of underestimators). Moreover, a significantly greater proportion of underestimators believed that ‘a large stomach is a sign of good health and wellbeing’ compared to those who correctly estimated (40.7% vs. 5.9%, respectively, *p* < 0.001). However, both groups who underestimated or correctly estimated their weight did not believe that being overweight improves longevity of life (77.2% and 86.8%, respectively).

### 3.4. Accuracy of Weight Estimation in Relation to Socio-Demographic Variables, BMI and Waist Circumference

Binary logistic regression assessed the effects of socio-demographic characteristics, BMI and waist circumference on the accuracy of weight estimation (Table 4). Model 1 describes the effect of socio-demographic factors including age, education, employment status and area of residence. Models 2, 3 and 4 examine the effects of body mass and shape by including BMI and/or waist circumference, to assess whether current body weight status and shape (abdominal obesity) are a stronger predictor of underestimation than socio-demographic factors. Women from the urban sample were less likely to underestimate their weight compared with women living in the rural setting (Model 1; OR: 0.46; 95% CI 0.23–0.98). However, age, education and employment status were not associated with accuracy of weight estimation. Addition of BMI and/or waist circumference to the model had no effect on the association between accuracy of weight estimation and socio-demographic variables (Models 2–4). It was observed that being either overweight or obese increased the risk of underestimation (Model 2; OR: 18.63, 95% CI: 7.72–44.91; OR: 24.22; 95% CI: 7.40–79.26, respectively). A large waist circumference had a variable effect on accuracy, where alone it appeared to decrease likelihood (Model 3; OR: 0.30; 95% CI: 0.14–0.65) but with the addition of BMI increased likelihood of underestimation (Model 4; OR: 3.31; 95% CI: 1.07–10.03).

## 4. Discussion

This study suggests that in the area of northern Peru, an upper middle-income country undergoing considerable dietary and demographic change, there is high incongruity between self-perceived and measured weight status among women. A high level of underestimation in a group of women where excess weight prevalence reached almost two-thirds of the population was reported. Almost 71% of participants underestimated their body weight, and no incidence of overestimation was reported. In addition, underestimation of body weight was more common among overweight (90.0%) and obese (90.0%) than normal weight women (33.8%; *p* < 0.001, Table 2). These findings are in agreement with the results of a previous study in Peruvian cities, which found that one-third of normal weight participants considered themselves as underweight, and 38.0% and 89.0% of overweight and obese women, respectively, underestimated their weight [15]. A similar tendency to consistently underestimate body weight was also reported in a study of Peruvian rural, urban and rural to urban migrants, where 53.8% of the sample population underestimated their weight and no participant reported themselves as obese, despite a measured prevalence of obesity of 20.1% [14]. Research across other low and upper middle-income countries undergoing similar transitions show comparable findings, including in Sri Lanka, where only 24.7% of adults correctly predicted their weight close to measured weight [26]; in Nigeria, where 53.6% of adults underestimated their weight [27]; in Mauritius, which reported 40.8% underestimation among overweight or obese adults [28]. The observed high level of underestimation of body weight status across different BMI categories may indicate a shift in perspective as to what is considered a ‘normal’ and healthy weight. Indeed, evidence documents that this dynamic change in perception may occur when the majority of the reference group to whom individuals compare their weight become overweight or obese [29]. Evidence has also shown that socio-cultural factors contribute to a tendency to underestimate body weight status, including that women of Latin American background may hold a more positive attitude towards obesity [12,30]. This is mirrored by this study, which demonstrated that 45% of women who underestimated their weight status considered a large stomach being related to better wellbeing and positive health outcomes. However, multiple regression analysis suggested that BMI is a stronger predictor of weight status underestimation than waist circumference.

This study showed that underestimation of weight status was prevalent, especially amongst overweight and obese women. The ability to accurately perceive bodyweight and identify changes in ones’ bodyweight is recognised as essential to attenuate and prevent weight gain [31] and those who misperceive their weight show a lower awareness of associated risk factors and are overly optimistic beliefs about personal health [12]. This group may therefore be less likely to adopt appropriate behaviours such as healthy eating and physical exercise and engage less with health care services which encourage lifestyle modification to maintain and/or reduce body weight [32]. A high level of underestimation indicates low risk perception and low likelihood of recognising the health risks associated with excess weight [11]. In this study, a greater proportion of those who underestimated said they did not experience problems with controlling their weight (51.2% vs. 26.5%, *p* < 0.001) and may therefore not have been making attempts to control it, perhaps because of a low awareness that their weight is an issue—either in terms of health or image, or both. Furthermore, the results indicated that participants who underestimated their weight status had the highest level of disagreement or ambivalence regarding responsibility toward health and weight. In addition, 59.3% of them felt comfortable with their current weight compared to 20.6% of women who correctly estimated their weight status. It is worth mentioning that although only 10.0% of obese women were able to correctly estimate their weight status, 40.0% were at least able to recognise that their weight status was higher than ‘normal’ body weight. This may result from a genuine physical discomfort and/or increase in associated health issues, but may also reflect a changing awareness, acceptance and valuation of adiposity, which occurs in countries as they move through the transition, and factors such as media messaging, regulatory policies and health care promotion and access also change [33].

Consistent with results of previous studies [14], we found that living in urban areas was related to a lower probability of underestimation. This may be due to a growing level of acculturation within the urban population [12], who may be more exposed to ‘Western’ cultural ‘norms’, or an increasing awareness of the negatives of excess weight due to expanding media and health coverage [34]. These results showed no association of underestimation of weight status with age, educational level and employment status. While other studies have reported relationships between these factors and body weight misperception, results have been largely inconsistent [14,35].

### *Limitations* 

Despite the random sampling frame employed in the Moyobamba, the purposeful non-random sampling in the Yantaló group means that results may not be generalisable at the population level. Nevertheless, the sampling frame largely covered the boundaries of the village and is likely substantially representative of that region. Participants were not excluded on any basis other than gender and age, reducing the possibility of selection bias. Secondly, medical terminology for the terms ‘overweight’ and ‘obese’ have a different and often unclear connotation outside of the medical sphere and in areas where there are very limited instances of measuring body weight [36]. Thus, cultural-specific interpretations of the terminology may have influenced self-perceptions, where the two terms ‘overweight’ (sobrepeso) and ‘obese’ (obeso) may have been regarded as tantamount, or where obesity was seen as an ‘extreme’ size, an actual misunderstanding of the physical manifestations of these terms may have influenced self-assessment and use of the 10-point scale to assess weight as therefore unsuitable. The use of a scale to assess ones’ own weight bears difficulty in visualisation and thus estimation. The scale may hold advantages with findings that descriptive terminology may be seen as disagreeable. However, the use of silhouettes may have been more appropriate given the rural study environment where exposure to medical health terminology was low [12]. The survey question which asked about the importance of health and weight might have benefitted from asking these separately, given that the two components might have very different meanings, valuations and interpretations. Finally, the cross-sectional study design cannot confirm the direction of any association between current weight status and weight misperception. A longitudinal study, which captures weight variation and weight perceptions in the context of dietary, socio-economic and demographic change, would add valuable detail in describing the dynamics of place and time and the cause and effect of multiple variables.

## 5. Conclusions

This study is the first attempt to describe self-perception of weight and BMI in both rural and urban populations in an under-researched part of the Peruvian Amazon undergoing considerable epidemiological change. The modest sample size and different methodologies provide the opportunity to explore an important regional and national trend and are a good base for future research in this area. The results add to the scarce literature on weight misperception and its determinants in upper middle-income countries and suggest that in a society undergoing a rapid economic and nutrition transition where obesity is highly prevalent, misperception of weight is commonplace. Underestimation of weight occurred across all BMI categories, although the highest underestimation occurred among overweight participants. This highlights that underestimation may be a significant barrier to overcome for public health interventions, which may not be effective when the target population does not perceive their weight to be a problem. Furthermore, the strong cultural beliefs that a large body size is a sign of good overall health and wellbeing may counteract any weight-loss interventions predicated on health grounds. In contrast, the finding that urban living is associated with reduced likelihood of underestimation offers valuable insights and potential opportunities for intervention. The result of this study may inform future public health programs, which need to be culturally sensitive, appreciating that culture is an integral part of an individual’s self-perception and wellbeing. Future research in this area should further determine the factors affecting misperception of body weight in middle-income countries in order to develop appropriately tailored obesity prevention and control interventions.

## Figures and Tables

**Table 1:** Participant demographic characteristics.

	Urban (*n* = 114)		Rural (*n* = 116)		Total (*N* = 230)	
	*n*	*%*	*n*	*%*	*n*	*%*
Age ^a^						
18–29	57	50.0	34	29.3	91	39.6
30–40	25	21.9	27	23.3	52	22.6
41–51	13	11.4	30	25.9	43	18.7
52–64	14	12.3	14	12.1	28	12.2
≥65	5	4.4	11	9.5	16	7.0
Education ^a^						
None/Primary incomplete	13	11.4	39	33.6	52	22.6
Primary/Secondary incomplete	25	21.9	53	45.7	78	33.9
Secondary	38	33.3	19	16.4	57	24.8
Technical career/apprenticeship	38	33.3	5	4.3	43	18.7
Employment status ^a^						
Employed/Self-employed	77	67.5	34	29.3	111	48.3
Housewife	34	29.8	78	67.2	112	48.7
Retired	1	0.9	3	2.6	4	1.7
Unemployed	2	1.8	1	0.9	3	1.3
Civil status						
Married/Living with partner	73	64.0	87	75.0	160	69.6
Single	37	32.5	23	19.8	60	26.0
Divorced	1	0.9	1	0.9	2	0.9
Widowed	3	2.6	5	4.3	8	3.5
Number in household						
1−2	22	19.3	20	17.2	42	18.3
3−5	68	59.6	65	56.0	133	57.8
6+	24	21.1	31	26.7	55	23.9
Waist circumference (cm)						
≤80	27	23.7	16	13.8	43	18.7
≥80	87	76.3	100	86.2	187	81.3
Body mass index (BMI, kg/m^2^)						
Normal (18.0–24.9)	47	41.2	33	28.4	80	34.8
Overweight (25.0–29.9)	43	37.7	57	49.1	100	43.5
Obese (total) (30.0–39.9)	24	21.1	26	22.4	50	21.7
Obese grade 1 (30.0–34.9)	17	14.9	22	19.0	39	17.0
Obese grade 2 (35.0–39.9)	7	6.1	4	3.4	11	4.8
Self–perceived weight						
Underweight	13	11.4	16	13.8	29	12.6
Normal weight	82	71.9	84	72.4	166	71.9
Overweight/obese	19	16.7	16	13.8	35	15.2
Accuracy of weight estimation ^a^						
Underestimated weight	93	60.5	69	80.2	162	70.4
Correctly perceived weight	45	39.5	23	19.8	68	29.6.
Overestimated weight	0	0.0	0	0.0	0	0.0

^a^*p* < 0.05.

**Table 2:** Association between participants’ self-perceived weight and measured body mass index.

	BMI Category					
	Normal Weight		Overweight		Obese	
	*n*	*%*	*n*	*%*	*n*	*%*
Self-perceived weight ^a^						
Underweight	27	33.8	0	0.0	2	4.0
Normal weight	53	66.2	90	90.0	23	46.0
Overweight	0	0.0	10	10.0	20	40.0
Obese	0	0.0	0	0.0	5	10.0
Accuracy of weight estimation ^a^						
Underestimated	27	33.8	90	90.0	45	90.0
Correctly estimated	53	66.2	10	10.0	5	10.0
Overestimated	0	0.0	0	0.0	0	0.0

^a^*p* < 0.001.

**Table 3:** Associations between survey responses and accuracy of weight estimation.

	Accuracy of Weight Estimation			
	Underestimated Weight		Correctly Estimated Weight	
	*n*	*%*	*n*	*%*
*My health and my weight are very important to me*				
Yes	132	81.5	62	91.2
No	2	1.2	0	0.0
Slightly/A bit	28	17.3	6	8.8
*It’s my responsibility to look after my weight* ^1^				
Yes	56	34.6	66	97.1
No	50	30.9	0	0.0
Slightly/A bit	56	34.6	2	2.0
*I feel comfortable with my weight* ^1^				
Yes	96	59.3	14	20.6
No	46	28.4	33	48.5
Slightly/A bit	20	12.3	21	30.9
*I often have problems controlling my weight* ^1^				
Yes	43	26.6	37	54.4
No	83	51.2	18	26.5
Slightly/A bit	36	22.2	13	19.1
*A large stomach is a sign of good health and wellbeing* ^1^				
Yes	66	40.7	4	5.9
No	69	42.6	64	94.1
Slightly/A bit	27	16.7	0	0.0
*In my opinion, overweight makes people live longer than thin people*				
Yes	12	7.4	2	2.9
No	125	77.2	59	86.8
Slightly/A bit	25	115.4	7	10.3

N, number, ^1^ *p* < 0.001.

**Table 4:** Logistic regression models assessing association between accuracy of weight estimation and socio-demographic variables, body mass index and waist circumference.

	Model 1	Model 2	Model 3	Model 4
	OR (95% CI)	OR (95% CI)	OR (95% CI)	OR (95% CI)
Age				
18–29 (Ref)	1	1	1	1
30–41	3.38 (1.35–8.48)	2.10 (0.69–6.40)	2.88 (1.12–7.41)	2.23 (0.73–6.81)
42–51	2.09 (0.78–5.59)	0.87 (0.26–2.89)	1.53 (0.55–4.26)	1.13 (0.31–4.07)
52–64	1.09 (0.38–3.09)	0.41 (0.11–1.53)	0.77 (0.26–2.26)	0.50 (0.13–1.98)
≥65	2.48 (0.59–10.41)	1.86 (0.34–10.23)	1.80 (0.42–7.68)	2.70 (0.42–17.35)
Education level				
None/primary incomplete (Ref)	1	1	1	1
Primary	0.61 (0.49–3.31)	1.10 (0.35–3.42)	1.15 (0.44–3.01)	1.16 (0.35–3.83)
Secondary/primary incomplete	0.49 (0.24–1.99)	0.54 (0.15–1.99)	0.67 (0.23–1.97)	0.51 (0.14–1.90)
Technical career/degree	0.98 (0.31–3.33)	1.31 (0.30–5.67)	1.12 (0.33–3.81)	1.14 (0.26–5.07)
Employment status				
Employed/self-employed (Ref)	1	1	1	1
Housewife	1.03 (0.51–2.12)	0.85 (0.35–2.06)	0.92 (0.44–1.93)	0.94 (0.38–2.36)
Retired/unemployed	1.17 (0.19–7.02)	1.55 (0.17–14.26)	1.07 (0.18–6.36)	1.96 (0.16–23.49)
Area of residence				
Rural (Ref)	1	1	1	1
Urban	0.46 (0.23–0.98)	0.38 (0.15–0.94)	0.45 (0.21–0.94)	0.39 (0.15–1.01)
BMI				
Normal weight (Ref)		1		1
Overweight		18.63 (7.72–44.91)		34.24 (11.55–101.45)
Obese		24.22 (7.40–79.26)		42.06 (11.17–158.32)
Waist circumference				
<80 cm (Ref)			1	1
≥80 cm			0.30 (0.14–0.65)	3.31 (1.07–10.03)

CI, confidence intervals. OR, odds ratio.

## References

[1] Hruby A., Manson J.A.E., Qi L., Malik V.S., Rimm E.B., Sun Q., Willett W.C., Hu F.B. (2016). Determinants and consequences of obesity. Am. J. Public Health.

[2] Di Cesare M., Bentham J., Stevens G.A., Zhou B., Danaei G., Lu Y., Bixby H., Hajifathalian K., Fortunato L., NCD Risk Factor Collaboration (2016). Trends in adult body-mass index in 200 countries from 1975 to 2014: A pooled analysis of 1698 population-based measurement studies with 19.2 million participants. Lancet.

[3] Fisberg M., Kovalskys I., Gómez G., Rigotti A., Cortés L.Y., Herrera-Cuenca M., Yépez M.C., Pareja V., Guajardo R.G., Zimberg I.Z. (2016). Latin American Study of Nutrition and Health (ELANS): Rationale and study design. BMC Public Health.

[4] Kelly T., Yang W., Chen C.S., Reynolds K., He J. (2008). Global burden of obesity in 2005 and projections to 2030. Int. J. Obes..

[5] Carrillo-Larco R.M., Jaime J., Gilman R.H., Checkley W., Smeeth L., Bernabe-Ortiz A., Bernabé-Ortizet A. (2018). Trajectories of body mass index and waist circumference in four Peruvian settings at different level of urbanisation: The CRONICAS Cohort Study. J. Epidemiol. Community Health.

[6] World Bank (2019). Data Catalogue: Gender Statistics.

[7] Caballero B., Vorkoper S., Anand N., Rivera J.A. (2017). Preventing childhood obesity in Latin America: An agenda for regional research and strategic partnerships. Obes. Rev..

[8] Smith L.C., Ramakrishnan U., Ndiaye A., Haddad L., Martorell R. (2003). The Importance of Women’s Status for Child Nutrition in Developing Countries.

[9] Poterico J.A., Stanojevic S., Ruiz-Grosso P., Bernabe-Ortiz A., Miranda J.J. (2012). The association between socioeconomic status and obesity in Peruvian women. Obesity.

[10] Swinburn B.A., Sacks G., Hall K.D., McPherson K., Finegood D.T., Moodie M.L., Gortmaker S.L. (2011). The global obesity pandemic: Shaped by global drivers and local environments. Lancet.

[11] Lemon S.C., Rosal M.C., Zapka J., Borg A., Andersen V. (2009). Contributions of weight perceptions to weight loss attempts: Differences by body mass index and gender. Body Image.

[12] Cachelin F., Monreal T., Juarez L. (2006). Body image and size perceptions of Mexican American women. Body Image.

[13] Araújo C., Dumith S., Menezes A., Hallal P. (2010). Measured weight, self-perceived weight, and associated factors in adolescents. Rev. Panam. Salud Publica.

[14] Loret de Mola C., Pillay T.D., Diez-Canseco F., Gilman R.H., Smeeth L., Miranda J.J. (2012). Body Mass Index and Self-Perception of Overweight and Obesity in Rural, Urban and Rural-to-Urban Migrants: PERU MIGRANT Study. PLoS ONE.

[15] Jacoby E., Goldstein J., López A., Núñez E., López T. (2003). Social class, family, and life-style factors associated with overweight and obesity among adults in Peruvian cities. Prev. Med..

[16] Kim S.Y., Herrman A., Song H., Lim T.S., Cramer E., Ahn S., Kim J., Ota H., Jim H.-J., Kim J. (2016). Exploring cultural differences in women’s body weight perception: The impact of self-construal on perceived overweight and engagement in health activities. Health Care Women Int..

[17] Kronenfeld L.W., Reba-Harrelson L., Von Holle A., Reyes M.L., Bulik C.M. (2010). Ethnic and racial differences in body size perception and satisfaction. Body Image.

[18] Creed-Kanashiro H.M., Bartolini R.M., Fukumoto M.N., Uribe T.G., Robert R.C., Bentley M.E. (2003). Formative research to develop a nutrition education intervention to improve dietary iron intake among women and adolescent girls through community kitchens in Lima, Peru. J. Nutr..

[19] Loret De Mola C., Quispe R., Valle G.A., Poterico J.A. (2014). Nutritional transition in children under five years and women of reproductive age: A 15-years trend analysis in Peru. PLoS ONE.

[20] Huayanay-Espinoza C.A., Quispe R., Poterico J.A., Carrillo-Larco R.M., Bazo-Alvarez J.C., Miranda J.J. (2017). Parity and Overweight/Obesity in Peruvian Women. Prev. Chronic. Dis..

[21] INEI (2017). Instituto Nacional de Estadística e Informática, Peru.

[22] Soriano R., De León Rosales S.P., García R., García-García E., Méndez J.P. (2012). High knowledge about obesity and its health risks, with the exception of cancer, among Mexican individuals. J. Cancer. Educ..

[23] World Health Organisation (1998). Obesity: Preventing and Managing the Global Epidemic.

[24] World Health Organizaton (2008). Waist Circumference and Waist-Hip Ratio: Report of a WHO Expert Consultation.

[25] Alberti K.G.M.M., Eckel R.H., Grundy S.M., Zimmet P.Z., Cleeman J.I., Donato K.A., Fruchart J.C., James W.P.T., Loria C.M., Smith S.C. (2009). Harmonizing the metabolic syndrome: A joint interim statement of the international diabetes federation task force on epidemiology and prevention; National heart, lung, and blood institute; American heart association; World heart federation; International. Circulation.

[26] Jayawardena R., Byrne N.M., Soares M.J., Katulanda P., Hills A.P. (2014). Body weight perception and weight loss practices among Sri Lankan adults. Obes. Res. Clin. Pract..

[27] Akindele M.O., Phillips J., Igumbor E., Useh U. (2017). Body Weight Misperception and Dissatisfaction Among Overweight and Obese Adult Nigerians. JMIR Public Health Surveill..

[28] Caleyachetty R., Kengne A.P., Muennig P., Rutter H., Echouffo-Tcheugui J.B. (2016). Misperception of body weight among overweight or obese adults in Mauritius. Obes. Res. Clin. Pract..

[29] Robinson E., Kirkham T.C. (2014). Is he a healthy weight? Exposure to obesity changes perception of the weight status of others. Int. J. Obes..

[30] Mama S.K., Quill B.E., Fernandez-Esquer M.E., Reese-Smith J.Y., Banda J.A., Lee R.E. (2011). Body image and physical activity among Latina and African American women. Ethn. Dis..

[31] Powell T.M., De Lemos J.A., Banks K., Ayers C.R., Rohatgi A., Khera A., McGuire D.K., Berry J.C., Albert M.A., Vega G.L. (2010). Body size misperception: A novel determinant in the obesity epidemic. Arch. Intern. Med..

[32] Wang Y., Liang H., Chen X. (2009). Measured body mass index, body weight perception, dissatisfaction and control practices in urban, low-income African American adolescents. BMC Public Health.

[33] Arantxa Colchero M., Caro-Vega Y., Kaufer-Horwitz M. (2014). Socioeconomic status and misperception of body mass index among Mexican adults. Salud. Publica. Mex..

[34] Gregory C.O., Blanck H.M., Gillespie C., Michele Maynard L., Serdula M.K. (2008). Health perceptions and demographic characteristics associated with underassessment of body weight. Obesity.

[35] Popkin B.M. (2015). Nutrition Transition and the Global Diabetes Epidemic. Curr. Diab. Rep..

[36] Ellis S., Rosenblum K., Miller A., Peterson K.E., Lumeng J.C. (2014). Meaning of the Terms “Overweight” and “Obese” Among Low-Income Women. J. Nutr. Educ. Behav..

